# Disease-specific mortality burdens in a rural Gambian population using verbal autopsy, 1998–2007

**DOI:** 10.3402/gha.v7.25598

**Published:** 2014-10-29

**Authors:** Momodou Jasseh, Stephen R. C. Howie, Pierre Gomez, Susana Scott, Anna Roca, Mamady Cham, Brian Greenwood, Tumani Corrah, Umberto D'Alessandro

**Affiliations:** 1 Medical Research Council, The Gambia Unit, Fajara, The Gambia; 2 INDEPTH Network, Accra, Ghana; 3 London School of Hygiene & Tropical Medicine, London, UK; 4 AFPRC General Hospital, Farafenni, The Gambia

**Keywords:** verbal autopsy, cause of death, mortality, disease-specific mortality, communicable disease, non-communicable disease, Farafenni, The Gambia

## Abstract

**Objective:**

To estimate and evaluate the cause-of-death structure and disease-specific mortality rates in a rural area of The Gambia as determined using the InterVA-4 model.

**Design:**

Deaths and person-years of observation were determined by age group for the population of the Farafenni Health and Demographic Surveillance area from January 1998 to December 2007. Causes of death were determined by verbal autopsy (VA) using the InterVA-4 model and ICD-10 disease classification. Assigned causes of death were classified into six broad groups: infectious and parasitic diseases; cancers; other non-communicable diseases; neonatal; maternal; and external causes. Poisson regression was used to estimate age and disease-specific mortality rates, and likelihood ratio tests were used to determine statistical significance.

**Results:**

A total of 3,203 deaths were recorded and VA administered for 2,275 (71%). All-age mortality declined from 15 per 1,000 person-years in 1998–2001 to 8 per 1,000 person-years in 2005–2007. Children aged 1–4 years registered the most marked (74%) decline from 27 to 7 per 1,000 person-years. Communicable diseases accounted for half (49.9%) of the deaths in all age groups, dominated by acute respiratory infections (ARI) (13.7%), malaria (12.9%) and pulmonary tuberculosis (10.2%). The leading causes of death among infants were ARI (5.59 per 1,000 person-years [95% CI: 4.38–7.15]) and malaria (4.11 per 1,000 person-years [95% CI: 3.09–5.47]). Mortality rates in children aged 1–4 years were 3.06 per 1,000 person-years (95% CI: 2.58–3.63) for malaria, and 1.05 per 1,000 person-years (95% CI: 0.79–1.41) for ARI. The HIV-related mortality rate in this age group was 1.17 per 1,000 person-years (95% CI: 0.89–1.54). Pulmonary tuberculosis and communicable diseases other than malaria, HIV/AIDS and ARI were the main killers of adults aged 15 years and over. Stroke-related mortality increased to become the leading cause of death among the elderly aged 60 years or more in 2005–2007.

**Conclusions:**

Mortality in the Farafenni HDSS area was dominated by communicable diseases. Malaria and ARI were the leading causes of death in the general population. In addition to these, diarrhoeal disease was a particularly important cause of death among children under 5 years of age, as was pulmonary tuberculosis among adults aged 15 years and above.

The development of a successful health care system should be supported by information on the frequency of specific diseases and their contribution to morbidity and mortality in the population. Therefore, measuring mortality, including cause-of-death determination, enhances every level of health care planning, resource allocation and service delivery systems (1, 2). Unfortunately, many countries with the greatest demand for this information have weak health systems that do not generate the requisite information to establish cause-of-death distributions (3, 4) as they also lack functional vital registration systems to capture all the deaths in their populations at specific periods (5). This is particularly true for sub-Saharan Africa where the majority of deaths occur at home with little or no chance of the cause of death being certified by qualified medical personnel. In this context, verbal autopsy (VA), despite its known limitations, is the most viable option for generating representative cause-of-death information.

The VA approach to determination of the cause of death is described in detail elsewhere (5). Used predominantly in research settings, the range of methods adopted for cause-of-death assignment includes physician-certification, data-derived algorithms, application of Bayes’ theorem, and direct statistical estimation of cause fractions (2). Physician-certified VA (PCVA) is an established method for assigning cause of death, but it is time consuming. Different approaches more suitable for resource-challenged settings have been developed (6). These include the InterVA, a computer-based automated VA coding model, and the adoption by WHO of a set of standardised questionnaires that facilitate comparison across different geographical and environmental settings (7). InterVA has been validated in different settings throughout its evolution (8–11).

The Gambia, like many other low-income countries, does not have a functional civil and vital registration system. Health facilities are also unable to provide such information. The only available information on causes of death derives from studies focused on specific target groups (mainly children aged under 5 years) (12–14) and from a hospital-based study in Banjul, representing a largely urban area (15). There are no data on the cause-of-death structure and its evolution over time at the national or sub-national level. However, relevant data have been collected routinely in a population of about 45,000 people in the town of Farafenni and its environs in the North Bank Region of The Gambia as part of the Farafenni Health and Demographic Surveillance System (HDSS). VAs conducted for deaths that occurred between 1998 and 2007 were used to evaluate the cause-of-death patterns and to establish the disease-specific mortality burdens for this part of The Gambia.

## Methods

### Study area

**Fig. 1:**
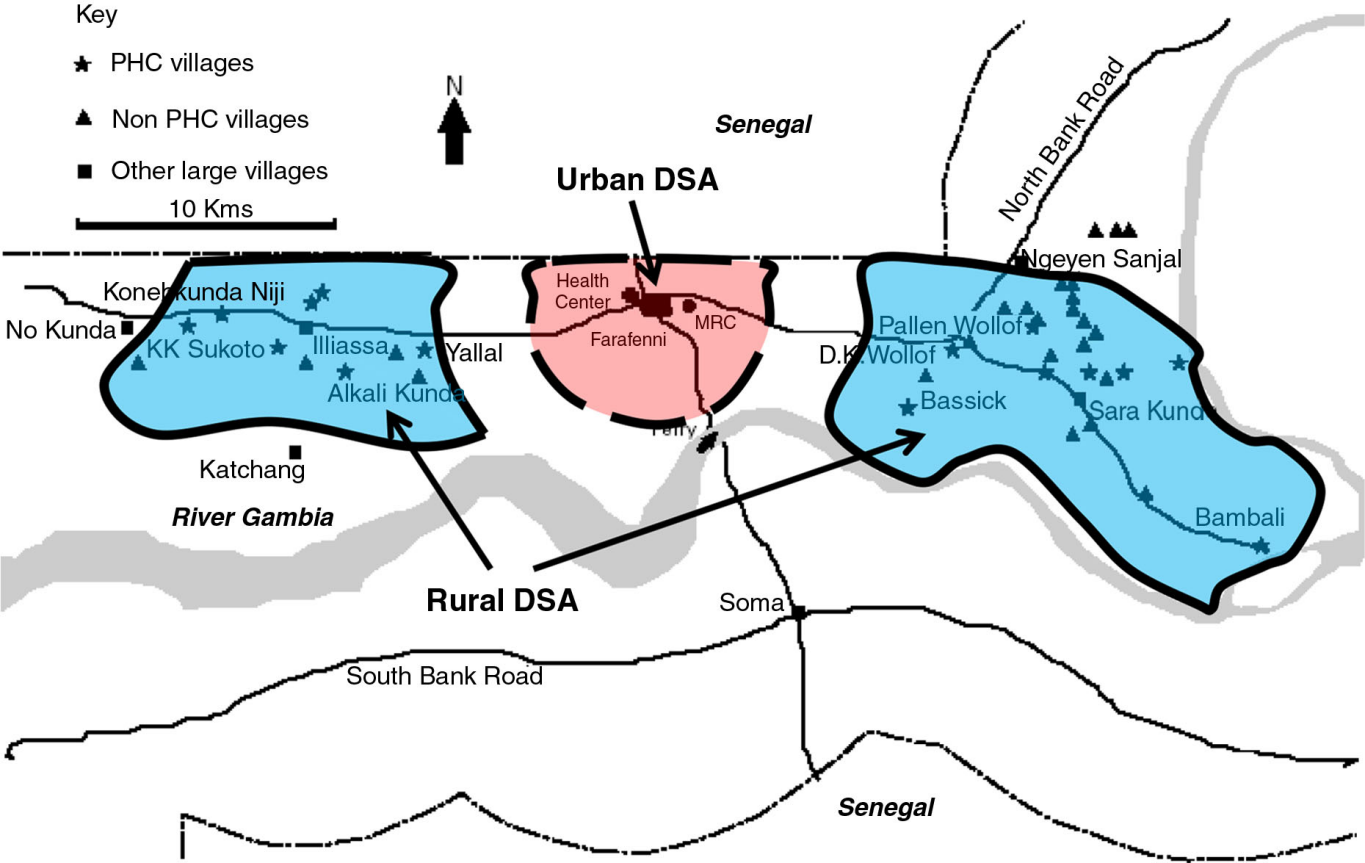
Location of the urban and contiguous rural areas east and west of Farafenni Town, which collectively constitute the Farafenni Health and Demographic Surveillance area.

The Farafenni HDSS was established in the North Bank Region of The Gambia in 1981 in two geographically contiguous clusters of 42 rural villages to the east and west of the town of Farafenni (population 25,000). In October 2002, the surveillance area was expanded to include Farafenni Town and 23 settlements within a 5-km radius of the town (see Fig. 1). The majority of the residents are subsistence farmers with very few earning salaries from employment. Detailed socio-demographic characteristics of the population are described elsewhere (13).

Malaria transmission is highly seasonal with most infections occurring between September and November. It was highly endemic in the past but has now declined substantially (16). The health care system consists of an under-resourced village-based primary health care system that has been operational since 1983 made up of 16 PHC Posts and five Dispensaries, a Health Centre for reproductive and child health services, and a 250-bed referral district hospital in the town of Farafenni, which was commissioned in 1999. Immunisation coverage levels for individual vaccines against childhood diseases are high (17), and use of ITNs is widespread (18). For the period 2000–2001, HIV prevalence among pregnant women in The Gambia was 1.0% for HIV-1 and 0.8% for HIV-2, and the corresponding levels for Farafenni were 0.4 and 0.3%, respectively (19). From a similar sentinel surveillance in 2008, the national prevalence levels were 1.6% for HIV1 and 0.4% for HIV2 (20). The majority of deaths occur at home and burials are held almost immediately or within a few hours after death, in line with religious beliefs. This constitutes a barrier to having deaths medically certified or being captured by vital registration.

### Data and statistical analysis

The surveillance procedures of the Farafenni HDSS are described in detail elsewhere (13, 21). The administration of VAs was introduced in 1998 for a sample of deaths and for specific study purposes. Routine administration of VA for every death in the area using the 2002 standard WHO VA questionnaires for neonates, children and adults commenced in 2005. VAs were administered retrospectively for deaths that occurred before 2005. VA interviews are conducted at least 40 days after death by trained fieldworkers, but the time lapse between death and interview has spanned up to 3 years in some cases. The main carers of the deceased persons prior to their deaths are targeted for the interviews, or if they are not available, other close relatives who were present during the period of illness and death. Interviews were conducted in one of the three main local languages spoken in the area.

The data collected using the three different age-specific questionnaires have been systematically transformed into the InterVA-4 input format and merged together. Responses for questions in the InterVA-4 format that could not be derived from the original data were coded as missing. InterVA-4 was applied with malaria prevalence set as ‘high’ and HIV prevalence as ‘low’, reflecting the situation in the study area for the period covered by this study and as recommended for West African regions. The data forms part of the INDEPTH Network pooled dataset for cause-of-death analyses covering Farafenni HDSS and 21 other INDEPTH HDSS sites (22).

The assigned causes of death were classified into six broad groups: infectious and parasitic diseases, cancers, other non-communicable diseases, neonatal, maternal, and external causes. In the analysis presented here, cause-specific mortality fractions (CSMFs) are derived from the three most likely causes of death and the residual indeterminate fraction assigned by InterVA-4 using disease codes in version 10 of the International Statistical Classification of Diseases and Related Health Problems, ICD-10. The output was merged with the detailed individual level data and analysed using STATA version 12 (Stata Corporation, College Station, TX, USA). Cause-specific mortality rates were calculated as the number of cause-specific deaths per 1,000 person-years of follow-up. They were derived using survival analysis techniques and Poisson regression, and individual deaths were associated with the most likely cause of death. Statistical significance at the 95% confidence level was determined using likelihood ratio tests. Since annual cause-specific mortality rates would not be meaningful due to small numbers of cause-specific deaths, the study period was divided up into three short periods with roughly similar numbers of deaths for the assessment of cause-specific mortality trends. These were defined *a priori* as 1998–2001, 2002–2004, and 2005–2007.

The Joint MRC/Gambia Government Ethics committee approved the establishment of the Farafenni HDSS and all instruments used to collect household and individual level information, including the VA questionnaires. Verbal consent was obtained for the administration of all VA questionnaires.

## Results

The mid-year population of the Farafenni HDSS in 1998 was 15,960 with a mean age of 23.0 years; and 44,644 in 2007 with a mean age 22.4 years. The age structure remained the same with 18% of the population aged less than 5 years in both periods; whilst the proportion aged 15 years and over was 53% in 1998 and 55% in 2007. Between 1 January 1998 and 31 December 2007, 3,203 deaths were recorded and VAs were administered for 2,275 (71%) of them. Annual VA coverage improved progressively from about 50% in the first 3 years of the study period and peaked at 87% in 2003. Coverage was generally high among adults aged 15 years or more throughout the study period. There was no difference in VA coverage by gender or area of residence.

Overall mortality declined by a third in the rural part of the demographic surveillance area over the 10-year period, a decline from 15 per 1,000 person-years in 1998–2001 to 10 per 1,000 person-years in 2005–2007 and by 12.5% in the urban part between 2002–2004 and 2005–2007, a decline from 8 to 7 per 1,000 person-years (Table 1). Mortality dropped by 55% among infants (from 78 to 35 per 1,000 person-years) and by 74% among children 1–4 years old (from 27 to 7 per 1,000 person-years). Overall mortality rates did not vary appreciably between ethnic groups in any of the three periods; and females had a lower mortality rate than males throughout.

**Table 1:** Number of deaths, proportion of deaths with verbal autopsy and death rates by period and population

	All			1998–2001			2002–2004			2005–2007		
				
	No. of deaths	Number of VAs conducted (% of deaths)	Death rate (per 1,000 PY)	No. of deaths	Number of VAs conducted (% of deaths)	Death rate (per 1,000 PY)	No. of deaths	Number of VAs conducted (% of deaths)	Death rate (per 1,000 PY)	No. of deaths	Number of VAs conducted (% of deaths)	Death rate (per 1,000 PY)
Age group												
<1 year	536	224 (42)	47	201	20 (10)	78	162	122 (75)	42	173	82 (47)	35
1–4 years	559	295 (53)	13	246	24 (10)	27	188	163 (87)	12	125	108 (86)	7
5–14 years	204	137 (67)	2	81	38 (47)	4	67	59 (88)	2	56	40 (71)	2
15–59 years	891	715 (80)	6	187	180 (96)	6	368	303 (82)	6	336	232 (69)	5
60+ years	1,013	904 (89)	58	287	280 (98)	63	359	331 (92)	59	367	293 (80)	54
Sex												
Male	1,727	1,217 (70)	12	531	287 (54)	17	609	522 (86)	11	587	408 (70)	9
Female	1,476	1,058 (72)	9	471	255 (54)	14	535	456 (85)	9	470	347 (74)	7
Ethnic group												
Wollof	1,192	825 (69)	10	349	172 (49)	15	418	355 (85)	9	425	298 (70)	8
Mandinka	1,262	950 (75)	11	453	272 (60)	16	428	386 (90)	11	381	292 (77)	9
Fula	629	416 (66)	9	194	95 (49)	14	239	195 (82)	10	196	126 (64)	7
Other	120	84 (70)	7	6	3 (50)	9	59	42 (71)	8	55	39 (71)	6
Area of residence												
Rural	2,145	1,491 (70)	13	1,002	542 (54)	15	635	572 (90)	12	508	377 (74)	10
Urban^a^	1,058	784 (74)	7				509	406 (80)	8	549	378 (69)	7
All	3,203	2,275 (71)	10	1,002	542 (54)	15	1,144	978 (85)	10	1,057	755 (71)	8

a Surveillance in this area started in October 2002.

CSMFs generated by InterVA-4 are presented in Table 2 by age group, sex, area of residence and ethnicity. Almost half (49.9%) of deaths over the 10-year period were attributed to communicable diseases. These were dominated by acute respiratory infections (ARI) (13.7%), malaria (12.9%) and pulmonary tuberculosis (10.2%). A similar pattern of cause-specific mortality proportions was maintained in all three periods. However, the proportion of HIV-related deaths showed an increasing trend over the three periods.

**Table 2:** Cause-specific mortality fractions (CSMFs) derived using recommended InterVA-4 settings by age group, period, sex, residence and ethnicity

Classification and causes of death	Total	<1 year	1–4 years	5–14 years	15–59 years	60+ years	1998–2001	2002–2004	2005–2007	Male	Female	Rural	Urban	Wollof	Mandinka	Fula	Other
Number of deaths	2,275	224	295	137	715	904	542	978	755	1,217	1,058	1,491	784	825	950	416	84
Infectious diseases																	
Malaria	12.9	15.8	38.3	28.3	7.5	5.9	10.7	14	12.9	12.3	13.5	13	12.6	15.4	10.3	13.5	13.9
ARI, including pneumonia	13.7	28.1	15.2	11.7	9.9	12.9	13.5	13.8	13.6	15	12.2	14	13	14.5	13.1	13.8	11.3
Pulmonary tuberculosis	10.2		0.4	1	14	14.3	10.2	9.7	10.8	12.6	7.4	9.6	11.3	7.4	12.5	8.8	17.5
HIV/AIDS-related	5.8	5.9	15	8.7	6.6	1.9	3.3	6.2	7.2	4.3	7.6	4.8	7.8	6.5	4.5	7.3	8
Diarrhoeal diseases	3.9	10.2	8.3	0.9	3.7	1.7	4.2	3.8	3.9	4	3.8	4.3	3.3	3.4	4.2	4.7	2.1
Meningitis and encephalitis	2.2	1.5	1.3	6.5	2.7	1.5	3	1.8	2	2.1	2.2	2.1	2.2	2.5	1.4	2.8	4.2
Other infectious diseases	1.2	0.1	0.1	2.8	1.6	1.2	1.1	1.4	1	1.7	0.6	1.2	1.2	0.9	1.2	1.6	0.7
Cancers																	
Digestive neoplasms	3.1				5.3	3.5	3	3	3.3	3.6	2.5	2.8	3.6	3.1	3.1	3.4	1.1
Respiratory neoplasms	1.6				1.6	2.7	2.3	1.5	1.3	2.4	0.7	1.8	1.3	0.8	2.2	1.5	3.3
Reproductive neoplasms	1.3				1.6	2.1	1.8	1.3	1	1	1.7	1.4	1.1	1.3	1.4	1.2	0.9
Other neoplasms	0.9				3.2	3.8	3	2.4	2.3	2.7	2.3	2.5	2.5	2.4	2.9	1.9	2.3
Other NCDs																	
Stroke	6.4				4.2	12.8	5.6	6.8	6.5	5.4	7.6	5.9	7.5	5.9	6.4	7.6	6.3
Acute abdomen	4.8	0.4	0.2	9.7	5.6	6	6.5	3.9	4.8	4.5	5.1	5	4.5	4.2	6.2	3.2	3.4
Severe malnutrition	1.6	3.4	6	1.5	0.2	0.8	0.8	1.7	2.1	1.6	1.5	1.5	1.9	2.1	1.4	1.3	0.9
Acute cardiac diseases	0.9				0.8	1.5	1	0.6	1	0.8	0.9	0.9	0.8	1.3	0.7	0.5	
Other cardiac diseases	1.3	0.5	0.8	2.2	1.1	1.7	1	1.5	1.4	1.3	1.3	1	2	1.6	1.2	1.1	1
Other unspecified NCDs	2.5	0.1	0.8	1.2	3	3.5	2.5	2.2	3	2.2	2.9	2.6	2.3	2.4	3.1	2	
Liver cirrhosis	2.5			1.8	0.8	1.3	0.5	0.9	1.1	1.1	0.7	1.1	0.6	1	0.5	1.6	1.2
Neonatal	2	20.6					1.4	3.2	0.9	1.5	2.6	2.9	0.4	2.1	2.1	1.6	2.4
External	2.5	0.7	1.2	5.9	3.8	1.7	2.9	2.1	2.6	2.7	2.2	2.4	2.5	2.2	2.9	2.2	1.8
Maternal	2.1				6.8		2.6	2.3	1.5		4.6	2	2.4	2.7	1.5	2.4	2.9
Indeterminate	16.6	12.8	12.4	17.8	16.2	19.1	19.1	15.9	15.8	17.1	16	17.3	15.3	16.5	17.2	16	14.7
Total	100	100	100	100	100	100	100	100	100	100	100	100	100	100	100	100	100

**Fig. 2:**
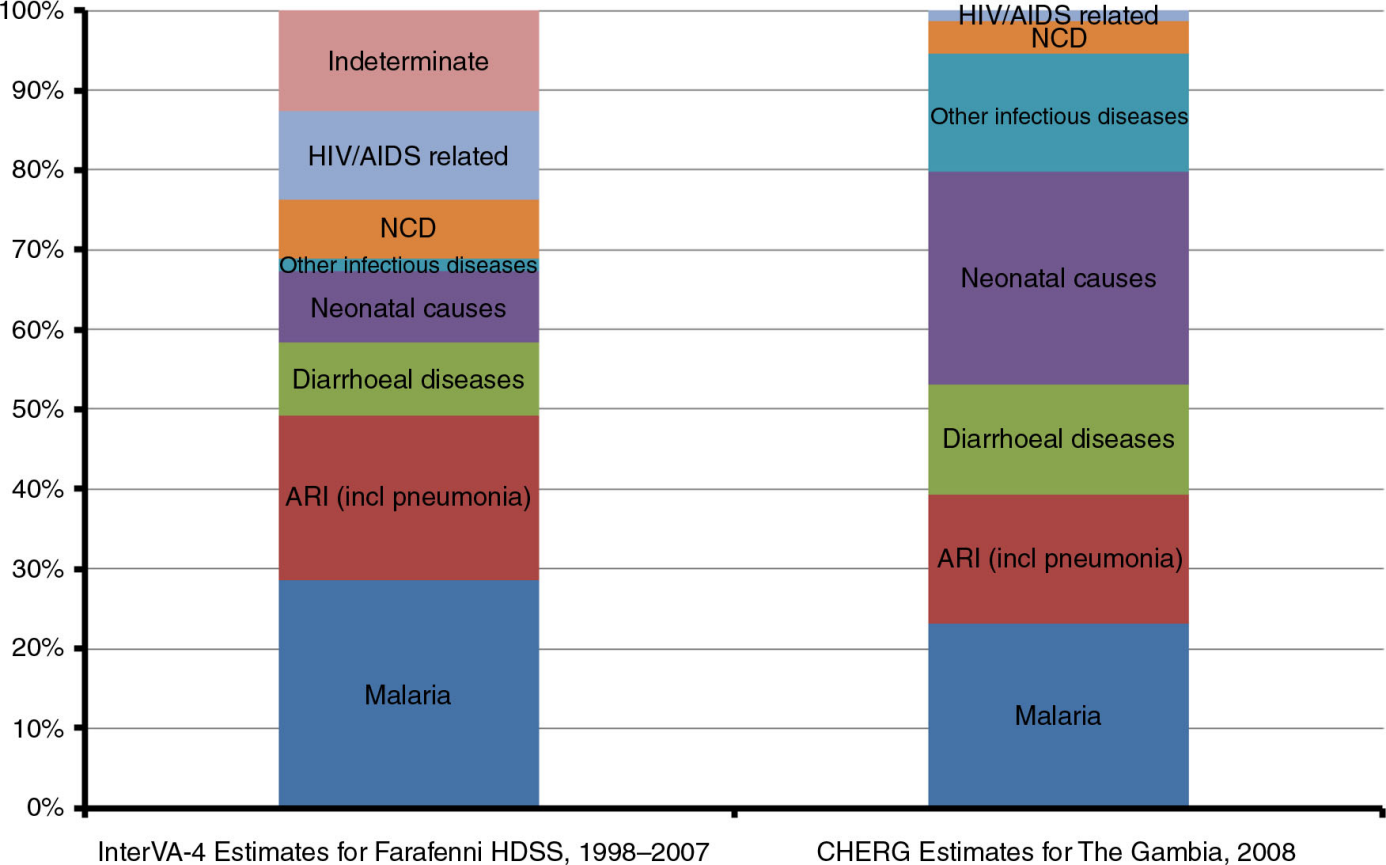
Comparison of the distribution of causes of death among children under 5 years of age by InterVA-4 for Farafenni HDSS, 1998–2007 and CHERG estimates for The Gambia, 2008 (27).

Malaria, ARI and diarrhoeal diseases accounted for more than half (58.5%) of deaths among children aged under 5 years (Fig. 2); 11% of deaths were attributed to HIV-related causes in this age group. More than a third (38%) of deaths among those aged 15–59 years were due to four communicable diseases: pulmonary tuberculosis (14%), ARI (including pneumonia) (9.9%), malaria (7.5%) and HIV/AIDS (6.5%). Overall, 46 and 37.8% of adult deaths were attributed to communicable and non-communicable diseases respectively.

Table 3 shows the top five cause-specific mortality rates for each age group and period. Overall, ARI, malaria, diarrhoeal diseases, neonatal sepsis, HIV/AIDS-related illnesses and severe malnutrition had the highest cause-specific mortality rates among children under 5 years of age. ARI mortality was consistently high among infants throughout the study period with an average rate of 5.59 per 1,000 person-years (95% CI: 4.38–7.15), followed by malaria with a rate of 4.11 per 1,000 person-years (95% CI: 3.09–5.47). The reverse pattern was observed among children aged 1–4 years who had a malaria mortality rate of 3.06 per 1,000 person-years (95% CI: 2.58–3.63) and an ARI mortality rate of 1.05 per 1,000 person-years (95% CI: 0.79–1.41). HIV-related mortality had the second highest rate in this age group (1.17 per 1,000 person-years [95% CI: 0.89–1.54]). Disease-specific mortality rates among children aged under 5 years increased overall between 1998–2001 and 2002–2004 due to increased VA coverage, but only significantly for malaria in infants (*p=*0.001) and children 1–4 years (*p<*0.0001). Although at comparatively lower levels, malaria, ARI, acute abdomen and HIV/AIDS constituted the greatest mortality burden among older children aged 5–14 years.

**Table 3:** Top five causes of death by age group and period

	1998–2007			1998–2001			2002–2004			2005–2007		
				
Age group and rank	Cause	No. of deaths	Cause-specific mortality rate per 1,000 PY (95% CI)	Cause	No. of deaths	Cause-specific mortality rate per 1,000 PY (95% CI)	Cause	No. of deaths	Cause-specific mortality rate per 1,000 PY (95% CI)	Cause	No. of deaths	Cause-specific mortality rate per 1,000 PY (95% CI)
<1 year												
1	ARI, including pneumonia	64	5.59 (4.38–7.15)	Neonatal sepsis	4	1.54 (0.58–4.11)	ARI, including pneumonia	36	9.34 (6.74–13.00)	ARI, including pneumonia	25	5.01 (3.38–7.41)
2	Malaria	47	4.11 (3.09–5.47)	Malaria	4	1.54 (0.58–4.11)	Malaria	21	5.45 (3.55–8.36)	Malaria	22	4.41 (2.90–6.69)
3	Neonatal sepsis	31	2.71 (1.91–3.85)	ARI, including pneumonia	3	1.16 (0.37–3.59)	Neonatal Sepsis	21	5.45 (3.55–8.36)	Diarrhoeal diseases	10	2.00 (1.08–3.72)
4	Diarrhoeal diseases	24	2.10 (1.41–3.13)	Neonatal pneumonia	2	0.77 (0.19–3.08)	Diarrhoeal diseases	12	3.11 (1.77–5.48)	Neonatal sepsis	6	1.20 (0.54–2.68)
5	HIV/AIDS-related	14	1.22 (0.72–2.07)	Other neonatal causes	2	0.77 (0.19–3.08)	Neonatal pneumonia	9	2.33 (1.21–4.49)	HIV/AIDS-related	6	1.20 (0.54–2.68)
	Indeterminate	11		Indeterminate	0		Indeterminate	7		Indeterminate	4	
1–4 years												
1	Malaria	131	3.06 (2.58–3.63)	Malaria	16	1.78 (1.09–2.91)	Malaria	78	4.92 (3.94–6.14)	Malaria	37	2.06 (1.49–2.84)
2	HIV/AIDS-related	50	1.17 (0.89–1.54)	Diarrhoeal diseases	2	0.22 (0.06–0.89)	HIV/AIDS-related	27	1.70 (1.17–2.48)	HIV/AIDS-related	23	1.28 (0.85–1.93)
3	ARI, including pneumonia	45	1.05 (0.79–1.41)	ARI, including pneumonia	1	0.11 (0.02–0.79)	ARI, including pneumonia	25	1.58 (1.06–2.33)	ARI, including pneumonia	19	1.06 (0.67–1.66)
4	Diarrhoeal diseases	26	0.61 (0.41–0.89)	Severe malnutrition	1	0.11 (0.02–0.79)	Diarrhoeal diseases	13	0.82 (0.48–1.41)	Diarrhoeal diseases	11	0.61 (0.34–1.11)
5	Severe malnutrition	18	0.42 (0.26–0.67)	Meningitis and encephalitis	1	0.11 (0.02–0.79)	Severe malnutrition	7	0.44 (0.21–0.93)	Severe malnutrition	10	0.56 (0.30–1.03)
	Indeterminate	9		Indeterminate	2		Indeterminate	4		Indeterminate	3	
5–14 years												
1	Malaria	47	0.53 (0.40–0.70)	Malaria	11	0.58 (0.32–1.05)	Malaria	25	0.76 (0.51–1.12)	Malaria	11	0.30 (0.17–0.54)
2	ARI, including pneumonia	16	0.18 (0.11–0.29)	Acute abdomen	6	0.32 (0.14–0.71)	HIV/AIDS-related	6	0.18 (0.08–0.41)	ARI, including pneumonia	6	0.16 (0.07–0.36)
3	Acute abdomen	15	0.17 (0.10–0.28)	ARI, including pneumonia	6	0.32 (0.14–0.71)	Acute abdomen	6	0.18 (0.08–0.41)	HIV/AIDS-related	6	0.16 (0.07–0.36)
4	HIV/AIDS-related	14	0.16 (0.09–0.27)	External causes	3	0.16 (0.05–0.49)	ARI, including pneumonia	4	0.12 (0.05–0.32)	Acute abdomen	3	0.08 (0.03–0.25)
5	External causes	9	0.10 (0.05–0.19)	Meningitis and encephalitis	3	0.16 (0.05–0.49)	External causes	3	0.09 (0.03–0.28)	Meningitis and encephalitis	3	0.08 (0.03–0.25)
	Indeterminate	8		Indeterminate	3		Indeterminate	4		Indeterminate	1	
15–59 years												
1	Pulmonary tuberculosis	107	0.68 (0.57–0.83)	ARI, including pneumonia	23	0.76 (0.50–1.14)	Pulmonary tuberculosis	44	0.75 (0.56–1.01)	Pulmonary tuberculosis	44	0.65 (0.49–0.88)
2	Other infectious diseases	107	0.68 (0.57–0.83)	Pulmonary tuberculosis	19	0.62 (0.40–0.98)	Other infectious diseases	44	0.75 (0.56–1.01)	Other infectious diseases	44	0.65 (0.49–0.88)
3	ARI, including pneumonia	78	0.50 (0.40–0.62)	Other infectious diseases	19	0.62 (0.40–0.98)	ARI, including pneumonia	37	0.63 (0.46–0.87)	Malaria	27	0.40 (0.27–0.58)
4	Malaria	61	0.39 (0.30–0.50)	Maternal causes^a^	18	0.59 (0.37–0.94)	Maternal causes^a^	25	0.43 (0.29–0.63)	HIV/AIDS-related	19	0.28 (0.18–0.44)
5	Maternal causes^a^	56	0.36 (0.28–0.47)	Acute abdomen	15	0.49 (0.30–0.82)	HIV/AIDS-related	24	0.41 (0.28–0.61)	ARI, including pneumonia	18	0.27 (0.17–0.42)
	Indeterminate	47		Indeterminate	9		Indeterminate	21		Indeterminate	17	
60+ years												
1	Pulmonary tuberculosis	137	7.84 (6.63–9.27)	ARI, including pneumonia	47	10.00 (7.79–14.00)	Stroke	59	9.62 (7.45–12.00)	Stroke	43	6.32 (4.69–8.52)
2	Other infectious diseases	137	7.84 (6.63–9.27)	Pulmonary tuberculosis	43	9.49 (7.04–13.00)	Other infectious diseases	54	8.80 (6.74–11.00)	ARI, including pneumonia	42	6.17 (4.56–8.35)
3	Stroke	134	7.67 (6.48–9.09)	Other infectious diseases	43	9.49 (7.04–13.00)	Pulmonary tuberculosis	54	8.80 (6.74–11.00)	Pulmonary tuberculosis	40	5.88 (4.31–8.02)
4	ARI, including pneumonia	130	7.44 (6.27–8.84)	Stroke	32	7.06 (4.99–9.99)	ARI, including pneumonia	41	6.68 (4.92–9.08)	Other infectious diseases	40	5.88 (4.31–8.02)
5	Malaria	63	3.61 (2.82–4.62)	Malaria	24	5.30 (3.55–7.90)	Acute abdomen	20	3.26 (2.10–5.05)	Acute abdomen	24	3.53 (2.36–5.26)
	Indeterminate	82		Indeterminate	28		Indeterminate	32		Indeterminate	22	

a Rate based on person-time contributed by women aged 15–49 years who constitute the population at risk of maternal causes of death.

Pulmonary tuberculosis, ARI, malaria and other infectious diseases (i.e. communicable diseases other than those listed in Table 2) were the main causes of death among adults aged 15–59 years. Maternity-related causes also had a relatively high mortality burden among women of reproductive age (15–49 years) (mortality rate of 0.36 per 1,000 person-years [95% CI: 0.28–0.47]). Overall, pulmonary tuberculosis and other infectious diseases were the main causes of death of adults in the Farafenni area during the 10-year period, each with a disease-specific mortality rate of 0.68 per 1,000 person-years (95% CI: 0.57–0.83). There was no significant change in their respective mortality rates in the latter two periods compared to the first (*p=*0.567 for 2002–2004 and 0.703 for 2005–2007 for TB; and *p=*0.276 and 0.885 for other infectious diseases for the same periods).

Pulmonary tuberculosis and other infectious diseases, stroke, ARI and malaria were the main causes of death among the elderly population aged 60 years and over. Mortality from stroke emerged in the last 6 years of the study period as the most important cause of death in this age group – 9.62 per 1,000 person-years in 2002–2004 (95% CI: 7.45–12.00) and 6.32 per 1,000 person-years in 2005–2008 (95% CI: 4.69–8.52).

The mortality burdens of both communicable and non-communicable diseases are summarised in Table 4 by period, age group, sex, ethnicity and area of residence. Mortality rates for both categories of disease dropped significantly in 2005–2007 compared with levels in 1998–2001. For all categories of disease, mortality burdens were greater in the rural than in the urban area. Males had a 42% (95% CI: 27–59%) and a 26% (95% CI: 9–47%) higher risk of dying from communicable and non-communicable diseases respectively compared to females. Mandinkas were more likely to die from a non-communicable disease than any other ethnic group (RR 1.45, 95% CI: 1.22–1.71). Cancer-related mortality was significantly higher among men than women (RR 1.66, 95% CI: 1.29–2.15) and among rural rather than urban residents (RR 1.73, 95% CI: 1.33–2.26). Only cancers arising from the reproductive system were more prevalent among females than males.

**Table 4:** Comparison of incidence of communicable and non-communicable diseases in the Farafenni demographic surveillance area by period and socio-demographic characteristics

			Non-communicable diseases					
			
	Communicable diseases		All		Cancers		Others	
				
	Rate ratio (95% CI)	*p*	Rate ratio (95% CI)	*p*	Rate ratio (95% CI)	*p*	Rate ratio (95% CI)	*p*
Period								
1998–2001	1		1		1		1	
2002–2004	1.09 (0.94–1.25)	0.265	0.91 (0.75–1.10)	0.317	0.85 (0.62–1.16)	0.310	0.94 (0.74–1.19)	0.625
2005–2007	0.75 (0.64–0.87)	<0.0001	0.67 (0.55–0.81)	<0.0001	0.59 (0.43–0.82)	0.002	0.71 (0.55–0.90)	0.006
Age group								
< 1 year	1		1				1	
1–4 years	0.45 (0.37–0.55)	<0.0001	0.64 (0.31–1.34)	0.238			0.64 (0.31–1.34)	0.238
5–14 years	0.08 (0.06–0.10)	<0.0001	0.34 (0.16–0.69)	0.003			0.30 (0.14–0.62)	0.001
15–60 years	0.17 (0.14–0.21)	<0.0001	1.64 (0.87–3.09)	0.126	1		0.90 (0.47–1.72)	0.750
60+ years	1.68 (1.40–2.03)	<0.0001	26.85 (14.34–50.28)	<0.0001	12.31 (9.53–15.91)	<0.0001	17.75 (9.44–33.36)	<0.0001
Sex								
Female	1		1		1		1	
Male	1.42 (1.27–1.59)	<0.0001	1.26 (1.09–1.47)	0.002	1.66 (1.29–2.15)	<0.0001	1.09 (0.91–1.31)	0.359
Ethnic group								
Wollof	1		1		1		1	
Mandinka	1.18 (1.04–1.33)	0.012	1.45 (1.22–1.71)	<0.0001	1.53 (1.15–2.05)	0.004	1.40 (1.14–1.72)	0.001
Fula	0.96 (0.82–1.12)	0.615	0.87 (0.70–1.09)	0.235	1.07 (0.74–1.53)	0.728	0.78 (0.59–1.03)	0.085
Other	0.84 (0.64–1.11)	0.232	0.56 (0.35–0.89)	0.015	0.82 (0.41–1.63)	0.562	0.44 (0.23–0.83)	0.011
Area of residence								
Urban	1		1		1		1	
Rural	1.59 (1.42–1.79)	<0.0001	1.56 (1.33–1.82)	<0.0001	1.73 (1.33–2.26)	<0.0001	1.47 (1.22–1.78)	<0.0001

## Discussion

There has been an appreciable decline in all-cause mortality in the Farafenni HDSS area over the 10-year period considered in this study. However, the pace of decline varied markedly between different age groups. Reduction in childhood mortality, especially among children aged between 1 and 4 years, accounted for much of the overall decline, similar to other countries in sub-Saharan Africa (23, 24). Improvements in the death rates for adults and the elderly were very modest.

With respect to causes of death, the results presented here give the first indication of the cause-specific pattern of mortality for all age groups over a period of time for any geographically defined population in The Gambia. Previous *ad hoc* investigations in the Farafenni HDSS area and other parts of the country used the physician coding method of VAs and focused on populations of interest such as children (12, 13, 25), women of reproductive age (26), or hospital patients (15). The outcomes of this study are therefore relevant from two perspectives. First, it provides an opportunity to evaluate the cause-of-death structure produced by the InterVA-4 model against the established disease epidemiology of the region and secondly it helps in defining the public health priorities of the area.

Using the recommended settings for malaria and HIV prevalence levels in West Africa, InterVA-4 has produced results that suggest three preventable and treatable diseases accounted for more than half (58%) of all deaths among children aged under 5 years between 1998 and 2007. These were malaria (28%), ARI (21%) and diarrhoeal diseases (9%) (Fig. 2). These proportions compare reasonably well with the distribution of under-5 deaths in 2008 estimated for The Gambia by the Child Health Epidemiology Reference Group (CHERG), with 53% of under-5 deaths being attributed to malaria, pneumonia and diarrhoeal diseases (27). These three diseases also dominate the overall cause-of-death structure compiled for children aged under 5 years in sub-Saharan Africa (27, 28). A similar study in Niakhar in central Senegal about 150 km north of the Farafenni HDSS, showed the same pattern of mortality burden among children under 5 years old for these three specific diseases (29), although malaria cases were included in a broader classification of fevers of unknown origin.

The InterVA-4 results suggest that only about one-tenth (9%) of under-5 deaths were due to neonatal causes, substantially lower than the CHERG estimate of 27% for The Gambia (Fig. 2). This is likely to be due to the relatively low VA coverage for infants during the study period; only a quarter (51/199) of neonatal deaths had a VA administered. Assuming that all neonatal deaths were due to neonatal causes, this will increase the proportion of under-5 deaths due to neonatal causes to 30%, which is similar to the CHERG estimate.

In view of the relatively low HIV prevalence in Farafenni, InterVA appears to have overestimated the proportion of under-5 deaths from HIV/AIDS, ranking it as the second highest killer among children aged 1–4 years (Table 3). Malnutrition is prevalent in rural Gambia and constitutes a major public health concern. About a quarter of children aged less than 5 years in the North Bank Region of The Gambia are underweight (18). It is therefore possible that the InterVA-4 model misinterpreted symptoms of malnourishment in children and returned the cause of their deaths as HIV-related, thus overestimating deaths resulting from HIV/AIDS. A similar conclusion was reached in another study in Kenya that compared the outcomes of an earlier version of InterVA and physician coding of the same VAs (11).

**Fig. 3:**
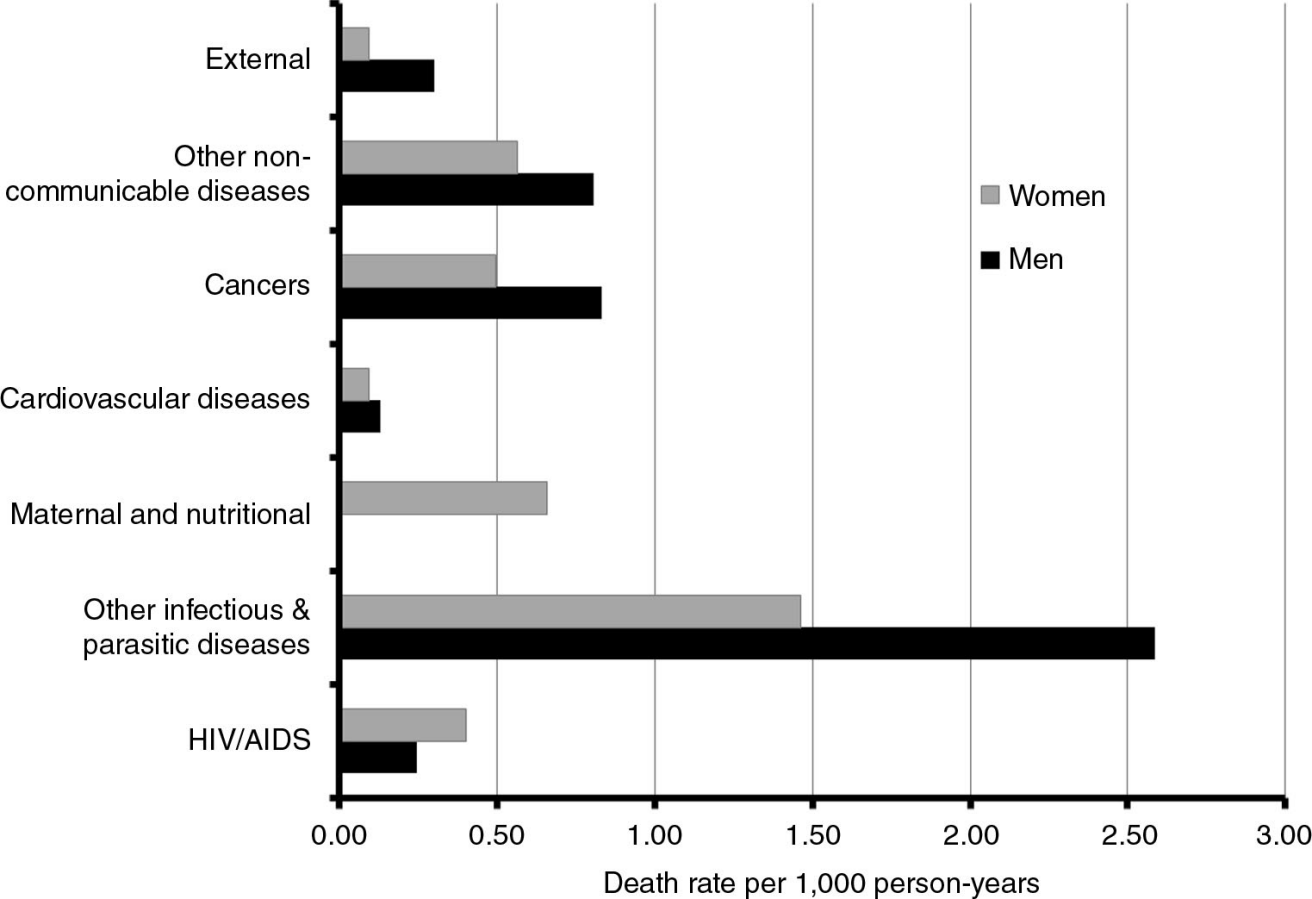
Cause and sex-specific mortality rates among Farafenni adults aged 15–59 years, 1998–2007.

The plausibility of the cause-specific mortality proportions for adults aged 15–59 years was assessed by re-grouping all causes by sex into the categories indicated in Fig. 3 and comparing the resulting patterns of cause and sex-specific mortality rates with those derived for the African region as a whole in 2008 (28). Apart from differences in the magnitudes of the burdens of HIV/AIDS and external (including injury) mortality, the InterVA-derived cause and sex-specific patterns of mortality among adults in the Farafenni HDSS area are remarkably similar to those of the African region. Common features between the two sets of mortality patterns include higher mortality rates among males for external, communicable and non-communicable disease-related causes of death and a higher HIV/AIDS mortality rate among females than among males. Communicable diseases, especially pulmonary tuberculosis, constitute the greatest mortality burden among adults aged 15–59 years in the Farafenni HDSS area.

As expected, there were more deaths among the elderly population aged 60 years and over, the group who had the highest VA coverage (89%). However, in the absence of an established cause-of-death structure for the elderly in an epidemiological setting such as that of the Farafenni HDSS, the plausibility of the disease-specific mortality burdens in this age group cannot be assessed with certainty. Judging from the reasonable cause-of-death distributions obtained for children and adults, and the fact that the InterVA-4 model has not returned any unexpected or unreasonable cause or causes of death in the elderly, the cause-of-death structure obtained can be accepted as a reasonable reflection of the pattern of mortality among the elderly in the area. The results show that stroke has emerged as the leading cause of death among this population in the latter part of the period considered. The rise in cardiovascular risk factors in this population to levels prevalent in the main urban centre of The Gambia was established between 1996 and 1997 (30). Hence, the level of stroke mortality produced by the model is plausible. Decreases in cause-specific mortality rates for this age group were not statistically significant and imply a stable cause-of-death pattern over the study period.

Despite using VA data covering less than three-quarters of all deaths in the period, especially among children aged under 5 years, and transforming these data from their original structure to meet InterVA-4 requirements, the model has proven useful in providing a population-based cause-of-death structure and disease-specific mortality rates for a sub-national population of The Gambia. The derived cause-specific mortality rates should be interpreted as the minimal possible rates because verbal autopsies were not administered for 29% of the deaths considered in the study, especially among children aged less than 5 years. In its Health Policy document for the period 2012–2020, The Gambia government identified the main causes of death in the country, albeit without strong supporting evidence, as malaria, pneumonia, anaemia, diarrhoeal diseases, road traffic accidents, pregnancy complications and cardiovascular diseases (20). Concern was also expressed over the increasing incidences of tuberculosis and HIV/AIDS. The results of this study reflect the same pattern of disease-specific mortality burden in the Farafenni HDSS area, and confirm the double burden of communicable and non-communicable diseases, which constitute a huge strain on a relatively weak local health care delivery system.

The InterVA-4 model should be subjected to further methodological tests and fine tuning to enhance its performance in an epidemiologically dynamic setting like that of the Farafenni HDSS area. Whilst an earlier study confirmed that the model is capable of detecting a significant epidemic of HIV-related mortality in South Africa (31), and that the use of VA tracked effectively an epidemiological transition in rural India (32), it has not been sufficiently validated in settings with changing malaria epidemiology and lower HIV prevalence than the regional level prescribed by the model. With the recent decline in malaria incidence in The Gambia (16, 33), a comparison of the results obtained from this study with those generated by setting the HIV and malaria prevalence levels that reflect the current epidemiological situation will show the extent to which death from communicable diseases will be redistributed. Non-communicable disease-related deaths will not be affected. The outcome of such a comparison will identify and document potential methodological limitations of the model in an environment like the Farafenni HDSS and facilitate future enhancement of the model.
